# Association Between Depressive Symptoms and Pain Intensity in Patients With Arthritis

**DOI:** 10.1002/brb3.71493

**Published:** 2026-05-18

**Authors:** Limei Jiang, Ming Tang, Yufan Zheng, Yao Zhang, Jing Li, Qingyue Kong, Fujie Jing

**Affiliations:** ^1^ Shandong University of Traditional Chinese Medicine Jinan China; ^2^ Shandong Provincial Hospital Affiliated to Shandong First Medical University Jinan China; ^3^ Affiliated Hospital of Shandong University of Traditional Chinese Medicine Jinan China; ^4^ Shandong College of Traditional Chinese Medicine Yantai China

**Keywords:** depressive symptoms, influencing factors, intensity of pain, patients with arthritis

## Abstract

**Background:**

Arthritis manifests with joint pain and stiffness that impair physical function and significantly elevate depression risk. However, the magnitude of pain's impact on depressive symptoms and related factors in arthritis patients warrants further investigation. Clarifying this association and its mechanisms is important for improving mental health and quality of life in this population.

**Study Design:**

This study analyzed data from the China Health and Retirement Longitudinal Study (CHARLS) in 2020, focusing on 1020 patients with arthritis.

**Methods:**

Depressive symptoms were assessed using the 10‐item Center for Epidemiologic Studies Depression Scale (CESD‐10), with a score ≥10 defining the presence of clinically significant depressive symptoms. Univariate analysis was performed using the Chi‐square test. A binary logistic regression model was employed to examine the association between pain intensity (binary: present/absent) and depressive symptoms, while controlling for multiple covariates, including demographics, health behaviors, and functional status.

**Results:**

Among the 1020 arthritis patients, the presence of pain significantly increased the risk of depressive symptoms (OR = 1.743, *p* = 0.002). Older age (OR = 0.982, *p* = 0.042), a higher educational level (e.g., high school or above: OR = 0.446, *p* = 0.005), better self‐rated health (OR = 0.408, *p* < 0.001), possessing internet use skills (OR = 0.625, *p* = 0.004), and retaining housework ability (OR = 0.466, *p* < 0.001) were identified as protective factors against depressive symptoms. Stratified analyses further revealed that the association between pain and depression was more pronounced among males, patients aged <65 years, and those with lower educational attainment.

**Conclusions:**

This study confirms that pain is an independent risk factor for depressive symptoms in arthritis patients, and this association exhibits heterogeneity across demographic subgroups. Higher educational attainment, positive self‐rated health, and maintenance of daily functional and social connectivity serve as psychological protective resources. Clinical practice should identify high‐risk patients and implement personalized strategies integrating pain management, psychological support, and functional promotion to alleviate depressive symptoms and enhance health.

## Graphical abstract

This study of 1,020 arthritis patients from CHARLS 2020 shows that pain significantly increases depressive symptom risk (OR = 1.743, p = 0.002). Older age, higher education, good self‐rated health, internet use, and retained housework ability are protective factors. The pain‐depression association is stronger among males, patients aged <65 years, and those with lower education.

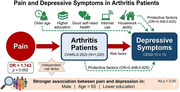

## 1 Introduction

Arthritis is a common chronic condition among the elderly (Yin et al. 2023), particularly affecting weight‐bearing joints such as the knees, hips, and hands (Hawker and King 2022; Lai et al. 2021). The pain associated with arthritis can significantly diminish patients' quality of life. Joints, as critical components of the musculoskeletal system, are essential for supporting body weight, maintaining balance, and facilitating various daily activities (Wang et al. 2023; Zeng et al. 2023). The health of these joints is crucial for preserving the independence of older adults (Zhai et al. 2023; Briggs et al. 2016). However, the pain caused by arthritis not only restricts their mobility but may also lead to a loss of independence, requiring them to rely on others for assistance with daily tasks, thereby having a profound negative impact on their overall quality of life (Simon et al. 2021; Woolf and Pfleger 2003).

Pain is one of the primary symptoms of arthritis, and it is particularly pronounced among the elderly (Messier et al. 2021). This pain not only poses a threat to patients' physical health but also significantly affects their mental well‐being (Wu et al. 2021). Research indicates that there is a close association between arthritis pain and depressive symptoms, with greater pain intensity correlating with more severe depressive symptoms (Griffin et al. 2022; Graham‐Engeland et al. 2016). This connection is especially evident among older adults, who typically face more health challenges and life difficulties, exacerbating the risk of fatigue and depression due to the persistent presence of pain (Hawker et al. 2011). Moreover, chronic pain induced by arthritis can lead to sleep disturbances (Irwin et al. 2023), further deteriorating patients' psychological health.

As the condition progresses, arthritis may result in joint deformity and functional loss, severely impacting patients' daily lives and mobility (Scanzello and Goldring 2012). The symptoms of this disease are often chronic and recurrent, causing long‐term physical discomfort and psychological stress for patients. Among the various types of pain, musculoskeletal pain, neuropathic pain, and visceral pain, which are commonly experienced by the elderly, have a particularly significant impact on depressive symptoms (Arcury et al. 2016). Studies have found that female elderly patients may experience more severe depression as a result of pain than their male counterparts. The severity of the pain largely determines the level of risk for depression (Dai et al. 2022). Given that depressive symptoms in elderly arthritis patients are often underestimated and inadequately treated, understanding the relationship between pain intensity, pain types, and depression, and implementing targeted interventions for elderly arthritis patients, is crucial for improving treatment outcomes.

This study aims to identify the depressive symptoms in arthritis patients and investigate the relationship between depression and pain intensity in this population. By determining the factors that influence depressive symptoms in arthritis patients, this research seeks to enhance long‐term pain management strategies, utilizing a comprehensive approach to alleviate depression and ultimately improve the quality of life for these patients.

## 2 Methods

### 2.1 Study Design

The study utilized data from the 2020 wave of the China Health and Retirement Longitudinal Study (CHARLS). This dataset employed a multistage probability sampling strategy. Zhao et al. (2014) provided a detailed description of the CHARLS design and sampling methods. In the first stage of sampling, 150 county‐level units across China were randomly selected. In the second stage, three primary sampling units were chosen from each county‐level unit, including both rural and urban areas. Ethical approval for CHARLS was obtained from the Biomedical Ethics Review Committee of Peking University (IRB00001052‐11015), and no additional ethical approval was required for this study (Zhao et al. 2014; Rong et al. 2020). Written informed consent was obtained from all participants before the interviews were conducted.

### 2.2 Sample and Setting

Arthritis is a condition characterized by inflammation within or around the joints, commonly triggered by factors such as joint wear and tear, abnormal immune responses, infections, or genetic predispositions (Millerand et al. 2019). The primary symptoms include joint pain, swelling, stiffness, and limited mobility, with symptoms often worsening in the morning or after prolonged periods of inactivity (Geneen et al. 2017). Based on this, the study selected a sample of 1020 arthritis patients. The inclusion criteria were as follows: (1) the patient had been diagnosed with arthritis, and (2) the patient had reported all relevant data on depressive symptoms in 2020.

## 3 Measures

### 3.1 Pain Intensity

The primary independent variable of this study was pain. It was assessed by the question: “Are you often troubled with body aches?” The original responses were recorded as an ordinal categorical variable: 1 = “None,” 2 = “A little,” 3 = “Somewhat,” 4 = “Quite a bit,” and 5 = “Very.” To enhance clinical interpretability and align with clinical practice that focuses on the presence or absence of pain, the variable was recoded into a binary form: respondents answering “None” were classified into the “No pain” group (coded as 0), while all other responses were combined into the “Pain present” group (coded as 1). It should be emphasized that although the wording of the question includes the adverb “often,” this ordinal response scale is commonly used to assess pain intensity. Therefore, in this paper, the construct is uniformly referred to as “pain”.

### 3.2 Depressive Symptoms

The dependent variable was depressive symptoms, measured using the 10‐item Center for Epidemiologic Studies Depression Scale (CESD‐10). Respondents rated the intensity of each symptom over the past week on a 4‐point scale ranging from 0 (<1 day) to 3 (5–7 days). The two positively worded items (“I felt hopeful about the future” and “I was happy”) were reverse scored. According to the established clinical cutoff, a total score of ≥10 was defined as indicating the presence of clinically significant depressive symptoms.

### 3.3 Covariates

Based on a review of the literature and the theoretical framework, multiple sets of covariates were included. Sociodemographic factors consisted of age (continuous variable), gender, educational level, marital status, and current residence. Health and functional status factors included self‐rated health, medical visits for arthritis in the past year, ability to use the internet, and ability to perform housework.

### 3.4 Statistical Analysis

All analyses were performed using Stata 14.0, with the statistical significance level set at a two‐sided *p* value < 0.05. In descriptive analyses, categorical variables were reported as frequencies and percentages, and continuous variables as means and standard deviations. Univariate analysis was conducted using the Chi‐square test.

In terms of the model‐building strategy, to clearly demonstrate the contribution of different variable blocks and to test the robustness of the core association, two sequential logistic regression models were constructed. Model 1 (the core model) included pain and all sociodemographic covariates. Model 2 (the full model) added health and functional status covariates to Model 1. All covariates were entered into the models simultaneously based on a priori knowledge, without variable selection based on statistical criteria.

Given that the original pain variable was ordinal, the primary analysis was estimated using an ordinal logistic regression (proportional odds model). Concurrently, to facilitate clinical interpretation and for subgroup analyses, pain was treated as a binary variable (present/absent) in binary logistic regression as the main reporting format. Supplementary analyses also treated pain in its original five‐category form.

To explore the heterogeneity of the association, pre‐specified subgroup analyses were conducted by stratifying the sample by gender, age group (<65 years vs. ≥65 years), and educational level (≤primary school] vs. middle school). Furthermore, two sensitivity analyses were performed to assess the robustness of the findings: treating the CESD‐10 total score as a continuous variable in a linear regression model and treating the original ordinal pain variable as a continuous predictor in a logistic regression model. Finally, variance inflation factors (VIFs) were used to assess multicollinearity in the models. All VIF values were below 2.5, indicating no serious multicollinearity.

## 4 Results

### 4.1 Sample Description

**FIGURE 1:**
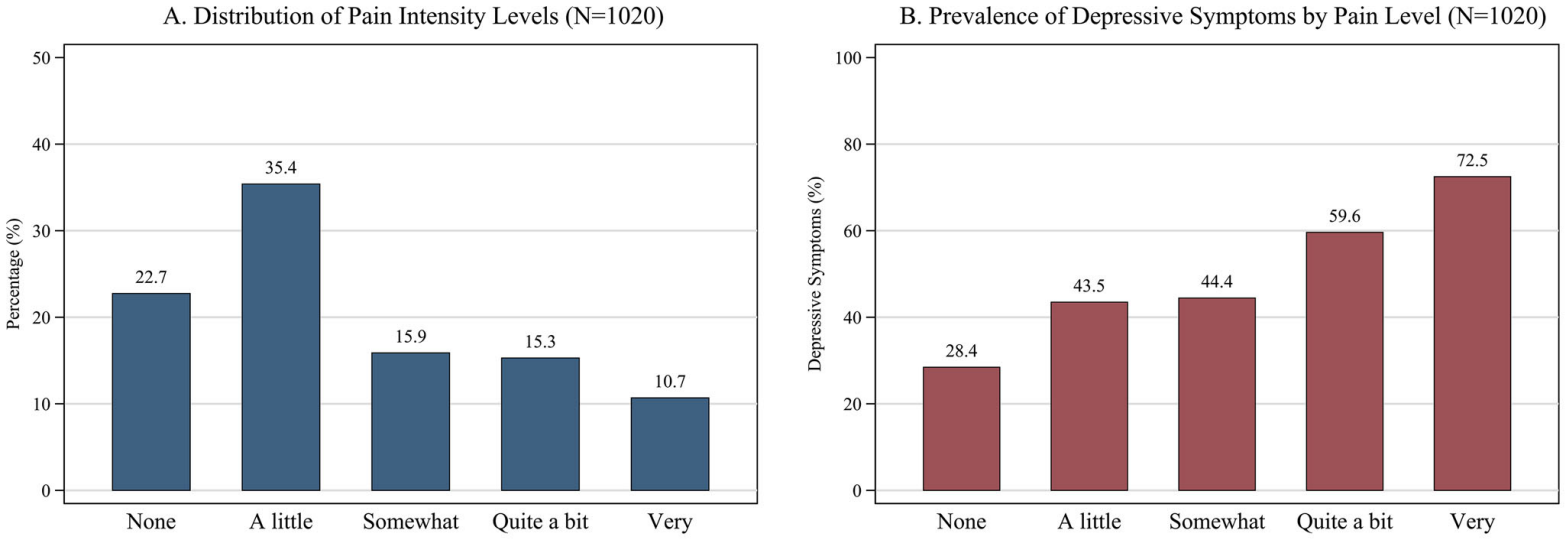
Pain intensity distribution and depression prevalence.

As shown in Figure 1, pain was highly prevalent in the studied population (N = 1020), with 77.3% reporting some degree of pain. A strong positive correlation was observed between pain intensity and depressive symptoms. Depressive symptom prevalence increased steadily from 43.5% in the “none” pain group to 72.5% in the “very” painful group, exceeding 50% once pain reached a moderate level. These findings suggest that pain severity is a significant indicator of depression risk. This highlights the clinical importance of integrated pain management and mental health screening, particularly for patients with moderate to severe pain.

Descriptive statistics regarding the depressive status of arthritis patients in relation to pain and other baseline characteristics are presented in Table 1. The results indicated that 45.78% of arthritis patients had depressive symptoms. Among these, patients with depressive symptoms reported a significantly higher mean pain level compared to those without depressive symptoms (2.92 vs. 2.25). Regarding general demographic information, a higher proportion of female arthritis patients had depressive symptoms (66.81%). Depressive symptoms were also more pronounced among patients with only a primary school education (46.04%). No significant relationship was found between depressive status and either marital status or place of residence.

**TABLE 1:** Descriptive statistics and univariate analysis results (*N* = 1020).

Variable	Not depressed *n* (%)	Depressed *n* (%)	*T*/*χ* ^2^	*p*
Pain intensity			36.36	0.000
No	166 (30.02)	66 (14.13)		
Yes	387 (69.98)	401 (85.87)		
Age [*N* (Mean)]	553 (60.53)	467 (60.46)	0.12	0.902
Gender			29.01	0.000
Male	276 (49.91)	155 (33.19)		
Female	277 (50.09)	312 (66.81)		
Education			39.48	0.000
None	89 (16.09)	130 (27.84)		
Primary school	230 (41.59)	215 (46.04)		
Middle school	144 (26.04)	87 (18.63)		
High school or above	90 (16.27)	35 (7.49)		
Marital status			2.56	0.109
Other	78 (14.10)	83 (17.77)		
Married	475 (85.90)	384 (82.23)		
Living place			7.78	0.005
Rural	304 (54.97)	297 (63.60)		
Urban	249 (45.03)	170 (36.40)		
Self‐rated health			83.23	0.000
Poor	111 (20.07)	219 (46.90)		
Good	442 (79.93)	248 (53.10)		
Recent medical experience			12.08	0.001
No	414 (74.86)	303 (64.88)		
Yes	139 (25.14)	164 (35.12)		
Internet skills			22.63	0.000
No	261 (47.20)	290 (62.10)		
Yes	292 (52.80)	177 (37.90)		
Housework skills			59.73	0.000
No	95 (17.18)	181 (38.76)		
Yes	458 (82.82)	286 (61.24)		

In terms of health status, 53.10% of arthritis patients with depressive symptoms rated their health as “good,” and 64.88% had not sought medical care for arthritis recently. Regarding behavioral factors, 62.10% of patients with depressive symptoms lacked internet use skills, while 61.24% retained the ability to perform housework.

### 4.2 Univariate Analysis Results

The univariate analysis of factors associated with depressive symptoms in arthritis patients is shown in Table 1. Pain was significantly associated with depressive symptoms (*p* < 0.05). Regarding demographic information, both gender and educational level showed significant correlations with the depressive status of arthritis patients (*p* < 0.05). In terms of health status, self‐rated health and recent medical visits for arthritis were significantly associated with depressive status (*p* < 0.05). Concerning lifestyle behaviors, both the ability to use the internet and the ability to perform housework were significantly correlated with depressive status (*p* < 0.05). Furthermore, marital status and place of residence showed no significant relationship with depressive symptoms.

### 4.3 Binary Logistic Regression Results

This study analyzed the influencing factors of depressive symptoms in arthritis patients using two sequential logistic regression models. In the baseline model (Table 2, Model 1), which controlled only for demographic variables, patients with bodily pain had 2.48 times the risk of developing depressive symptoms compared to those without pain (OR = 2.476, *p* < 0.001). When health and functional status variables were further included (Model 2), the effect size of pain was attenuated, but the association remained statistically significant and clinically meaningful (OR = 1.743, *p* = 0.002), indicating that pain is a robust independent risk factor for depressive symptoms. The attenuation of the effect size suggests that the impact of pain on depression is partially mediated indirectly through the deterioration of patients' self‐rated health and functional status.

**TABLE 2:** Logistic regression of depressive symptoms in arthritis patients (*N* = 1020).

Variable	Depressive symptom			
	Model 1		Model 2	
	OR (SE)	*p*	OR (SE)	*p*
Pain intensity	2.476 (0.414)	0.000	1.743 (0.311)	0.002
Age	0.998 (0.008)	0.778	0.982 (0.009)	0.042
Gender (ref: Male)	1.614 (0.231)	0.001	1.498 (0.229)	0.008
Education (ref: None)				
Primary school	0.787 (0.141)	0.180	0.853 (0.160)	0.399
Middle school	0.546 (0.119)	0.005	0.639 (0.147)	0.051
High school or above	0.350 (0.093)	0.000	0.446 (0.128)	0.005
Marital status (ref: Other)	0.852 (0.161)	0.397	0.793 (0.150)	0.170
Living place (ref: Rural)	0.823 (0.119)	0.180	0.914 (0.138)	0.551
Self‐rated health (ref: Poor)			0.408 (0.064)	0.000
Recent medical experience (ref: No)			1.314 (0.199)	0.072
Internet skills (ref: No)			0.625 (0.101)	0.004
Housework skills (ref: No)			0.466 (0.075)	0.000
Cons	0.635 (0.387)	0.456	7.991 (5.833)	0.004

The analysis of demographic factors revealed a clear pattern. The protective effect of age became apparent only after controlling for health and functional variables (Table 2, Model 2: OR = 0.982, *p* = 0.042). The risk of depression was significantly higher in female patients than in male patients (OR = 1.498, *p* = 0.008). Educational level exhibited a significant “dose–response” style protective gradient. Compared to the illiterate group, patients with a high school education or above had an approximately 55% lower risk of depression (OR = 0.446, *p* = 0.005). Marital status and place of residence were not significant in this study.

It is noteworthy that the newly added health and functional variables in Model 2 demonstrated strong protective effects. Among them, better self‐rated health (OR = 0.408, *p* < 0.001) and retained housework ability (OR = 0.466, *p* < 0.001) were the strongest modifiable factors associated with depressive symptoms, reducing the risk by approximately 59% and 53%, respectively. Possessing internet use skills was also a significant protective factor (OR = 0.625, *p* = 0.004). Recent medical visits showed a trend toward increased risk but did not reach conventional significance levels (OR = 1.314, *p* = 0.072).

### 4.4 Subgroup Analysis Results

#### 4.4.1 Subgroup Analysis by Gender

Table 3 presents the results of the logistic regression analysis stratified by gender. The gender‐stratified analysis revealed a difference in the impact of pain on depressive symptoms between male and female patients. Among male patients, those with pain had 2.45 times the risk of developing depressive symptoms compared to those without pain (OR = 2.452, *p* = 0.001), and this association was statistically significant. However, among female patients, the association between pain and depressive symptoms did not reach statistical significance (OR = 1.323, *p* = 0.263). This result indicates that the impact of pain on depressive symptoms is more pronounced in male arthritis patients.

**TABLE 3:** Stratified analysis by gender for factors associated with depressive symptoms in patients with arthritis (*N* = 1020).

Variable	Depressive symptom			
	Male		Female	
	OR (SE)	*p*	OR (SE)	*p*
Pain intensity	2.452 (0.659)	0.001	1.323 (0.311)	0.263
Constant	Yes		Yes	
Cons	1.354 (1.596)	0.797	34.817 (332.182)	0.000
*N*	431		589	

*Note*: Both models were adjusted for age, educational level, marital status, place of residence, self‐rated health, recent medical experience, internet skills, and housework skills.

Abbreviation: SE, standard error.

#### 4.4.2 Subgroup Analysis by Age

Table 4 presents the results of the logistic regression analysis stratified by age. Among the younger patient group (<65 years), the presence of pain was significantly associated with an increased risk of depressive symptoms (OR = 2.007, *p* = 0.002), indicating that younger patients with pain had approximately twice the risk of depression compared to those without pain. However, in the older patient group (≥65 years), the association between pain and depressive symptoms did not reach statistical significance (OR = 1.452, *p* = 0.238). This finding suggests that the impact of pain on depressive symptoms may vary by age, being more prominent and clearer in younger arthritis patients.

**TABLE 4:** Stratified analysis by age for factors associated with depressive symptoms in patients with arthritis (*N* = 1020).

Variable	Depressive symptom			
	<65 years		≥65 years	
	OR (SE)	*p*	OR (SE)	*p*
Pain intensity	2.007 (0.447)	0.002	1.452 (0.459)	0.238
Constant	Yes		Yes	
Cons	3.954 (1.865)	0.004	1.427 (0.750)	0.498
*N*	693		327	

*Note*: Both models were adjusted for gender, educational level, marital status, place of residence, self‐rated health, recent medical experience, internet skills, and housework skills.

Abbreviation: SE, standard error.

#### 4.4.3 Subgroup Analysis by Education

Table 5 presents the results of the logistic regression analysis stratified by educational level. Among patients in the lower education group (primary school or below), the presence of pain was significantly associated with an increased risk of depressive symptoms (OR = 1.844, *p* = 0.004), indicating a significantly elevated depression risk for low‐education patients experiencing pain. However, among patients in the higher education group (middle school or above), the association between pain and depressive symptoms did not reach statistical significance (OR = 1.650, *p* = 0.134). This finding suggests that educational level may have a potential moderating effect on the “pain‐depression” association, with higher education possibly acting as a protective factor that buffers the negative impact of pain on mental health.

**TABLE 5:** Stratified analysis by educational level for factors associated with depressive symptoms in patients with arthritis (*N* = 1020).

Variable	Depressive symptom			
	≤ Primary school		≥ Middle school	
	OR (SE)	*p*	OR (SE)	*p*
Pain intensity	1.844 (0.391)	0.004	1.650 (0.552)	0.134
Constant	Yes		Yes	
Cons	4.345 (3.785)	0.092	11.240 (13.064)	0.037
*N*	664		356	

*Note*: Both models were adjusted for age, gender, marital status, place of residence, self‐rated health, recent medical experience, internet skills, and housework skills.

Abbreviation: SE, standard error.

### 4.5 Sensitivity Analysis Results

Table 6 presents the results of two sensitivity analyses conducted to test the robustness of the main findings. First, when treating depressive symptoms (CESD‐10 total score) as a continuous outcome variable in a linear regression model, the effect of pain (binary variable) remained significant (Beta = 1.850, *p* < 0.001), indicating a stable positive effect of pain on the severity of depressive symptoms. Second, when treating the original ordinal pain variable (levels 1–5) as a continuous predictor in a logistic regression model, its association with depressive symptoms (binary variable) remained significant (OR = 1.247, *p* < 0.001), suggesting that each one‐level increase in pain was associated with approximately a 25% increase in depression risk. The results of both sensitivity analyses were consistent in direction and statistically significant compared to the main analysis, fully confirming the reliability of the core finding that “a positive association exists between pain and depressive symptoms.” All models were adjusted for the same set of covariates.

**TABLE 6:** Sensitivity analysis for the association between pain intensity and depressive symptoms in patients with arthritis (*N* = 1020).

Variable	Depressive symptom			
	Depressive symptoms as continuous outcome (Linear regression)		Pain intensity as continuous predictor (Logistic regression)	
	Beta (SE)	*p*	OR (SE)	*p*
Pain intensity	1.850 (0.446)	0.000	1.247 (0.074)	0.00
Constant	Yes		Yes	
Cons	17.344 (1.969)	0.000	6.513 (4.864)	0.012

*Note*: Both models were adjusted for age, gender, educational level, marital status, place of residence, self‐rated health, recent medical experience, internet skills, and housework skills. The left column presents results from a linear regression model where the CESD‐10 total score was treated as a continuous outcome (Beta coefficient reported). The right column presents results from a logistic regression model where the original ordinal pain intensity variable (levels 1–5) was treated as a continuous predictor.

Abbreviation: SE, standard error.

### 4.6 Statistical Diagnostics Analysis Results

Table 7 presents the multicollinearity diagnostic results for all predictor variables in the regression models. The VIF values for all variables ranged from 1.05 to 2.12, with a mean VIF of 1.40. As all VIF values were well below the commonly used empirical threshold of 5 (and the more stringent threshold of 10), this indicates that there was no serious multicollinearity problem in the constructed regression models. The low collinearity among predictors ensures the independence and stability of the effect estimates for each factor in the model, providing statistical support for the validity of the aforementioned main and stratified analysis results.

**TABLE 7:** Multicollinearity diagnostics for predictors in the logistic regression model (*N* = 1020).

Variable	VIF	1/VIF
Pain intensity	1.11	0.901
Age	1.40	0.716
Gender	1.19	0.841
Education		
Primary school	1.95	0.512
Middle school	2.12	0.473
High school or above	1.84	0.544
Marital status	1.15	0.866
Living place	1.23	0.810
Self‐rated health	1.16	0.860
Recent medical experience	1.05	0.954
Internet skills	1.40	0.711
Housework skills	1.16	0.863
Mean VIF	1.40	

Abbreviation: VIF, variance inflation factor.

## 5 Discussion

This study investigated the association between pain and depressive symptoms in arthritis patients, while examining the moderating and protective roles of sociodemographic and functional factors. Due to the cross‐sectional design, temporal causality cannot be established. It would be helpful to recommend future longitudinal analyses using subsequent CHARLS waves to explore whether pain intensity predicts future onset or persistence of depressive symptoms. The primary findings confirm that the presence of pain is a significant risk factor for depressive symptoms, and this association remained robust after adjusting for a series of health and functional variables (Vitaloni et al. 2019). More importantly, stratified analyses revealed heterogeneity in this association: it was more pronounced in patient subgroups including males, younger individuals (<65 years), and those with lower educational attainment (primary school or below) (Yan et al. 2023; Bawadi et al. 2021). Concurrently, better self‐rated health, retained housework ability, and internet use skills emerged as strong protective factors. These results not only corroborate the established consensus on the close link between pain and mental health but also provide deeper insights into the boundaries of individual and social resources that influence this relationship.

A significant positive association between pain and depressive symptoms in arthritis patients was found, which aligns with a substantial body of prior research (Whitlock et al. 2017). However, the model comparisons in this study offer important clues to elucidate the underlying mechanisms. The attenuation of the pain effect after including self‐rated health and functional variables suggests that pain's impact on depression is partially mediated by impairing patients' perception of their own health and diminishing their daily functional capacity (Guerra et al. 2024). This supports an integrative explanatory framework; chronic pain may not only directly affect brain regions involved in mood regulation via potential shared neuroinflammatory pathways but also exert broad psychosocial effects by triggering a secondary chain of emotion (Kim et al. 2021). Frequent pain experiences can erode a patient's sense of self‐efficacy, leading to frustration in accomplishing daily tasks. This accumulating sense of helplessness forms the core psychological foundation of depression (Cotroneo and Krasner 1977). Therefore, clinical interventions for arthritis patients should not be confined to analgesia alone but must simultaneously address the functional limitations and negative cognitive cycles triggered by pain.

The key protective factors identified in this study provide specific targets for tailored psychosocial interventions. Among them, educational level demonstrated a notable buffering effect. Stratified analyses revealed that the significant impact of pain on depression was concentrated primarily in the group with lower education. This strongly suggests that education, as an important psychosocial resource, may help individuals manage and cope with the challenges posed by pain more effectively by enhancing health literacy, problem‐solving skills, and the capacity to access support. This may be due to the fact that individuals with higher education levels typically possess better problem‐solving skills and stress management techniques, enabling them to approach and handle the challenges posed by their condition more rationally (Yu et al. 2022). Moreover, higher education is often associated with better socioeconomic status (Zhelenkova and Panichella 2023), allowing these patients to access superior medical resources and social support, thereby mitigating depressive symptoms. Furthermore, individuals with higher educational backgrounds are more likely to be informed about disease management and mental health, leading them to adopt healthier behaviors, such as regular exercise, balanced diets, and active participation in social activities, all of which contribute to alleviating depressive moods (Connell et al. 2016).

Meanwhile, those who rate their health as good, can use the internet, and can perform household chores experience significantly fewer depressive symptoms. These factors reflect the quality of life and sense of self‐efficacy in these patients. A positive self‐assessment of health indicates that patients have a favorable perception of their health status, which helps in reducing depressive moods. Patients who are able to use the internet generally have the ability to access information, allowing them to obtain relevant information on arthritis management, participate in online support groups, or receive telemedicine services, all of which can provide emotional support and practical coping strategies, thereby alleviating depressive symptoms (Wang et al. 2024). The ability to perform household chores indicates a level of independence and functional capacity in daily life, which can enhance self‐esteem and self‐efficacy (Gearhart 2023), thereby reducing the risk of depression. These factors are highly consistent in meaning with self‐rated health, the strongest protective factor as all reflect a positive appraisal of one's own condition and higher psychological resilience, serving as crucial psychological resources against depression.

However, arthritis patients with a history of medical consultations exhibit more severe depressive symptoms. This could reflect the challenges and pressures patients face when seeking medical help. For instance, frequent medical visits may increase patients' anxiety and uncertainty about their condition, particularly when treatment outcomes fall short of expectations, leading to feelings of disappointment and helplessness (Giacomini et al. 2012). Additionally, the financial burden, time consumption, and unequal access to medical resources during the healthcare process may exacerbate patients' psychological distress, worsening depressive symptoms (Adelman et al. 2014). This finding highlights the need for healthcare providers to pay close attention to the psychological well‐being of arthritis patients, in addition to addressing the physical treatment of the condition, by offering necessary psychological support and emotional care to help patients better cope with the psychological stress associated with their illness.

It is noteworthy that the association between pain and depression exhibited significant heterogeneity across different populations. This study found the association to be particularly prominent in male patients, whereas it did not reach significance in females. This may be related to social gender roles and norms of emotional expression, suggesting that psychological interventions for men should pay special attention to the linkage between their pain experience and emotional health (Dai et al. 2022). Furthermore, the association was significant in younger patients but attenuated in older patients. First, with age, patients may gradually adapt to the presence of pain and develop more effective coping strategies (Di et al. 2023). This finding may align with the adaptation and expectation theory, where older adults have higher psychological expectations for chronic conditions and pain, partially attributing them to normal aging, thereby reducing related psychological distress. In contrast, younger patients may experience stronger frustration as pain interferes with their core social roles in work and family. These findings partially support and partially challenge the existing literature, emphasizing the importance of focusing on within‐group differences when studying the mental health of populations with chronic conditions. Additionally, older individuals often have a more realistic and accepting attitude toward life, viewing the gradual decline in physical function as a natural part of aging (Wang et al. 2017), which alleviates anxiety and feelings of helplessness related to pain. Research also indicates that older adults are more likely to utilize social support networks to alleviate depressive moods (Bai and Cheng 2022), such as seeking help and support from family and friends, which may contribute to the reduction of depressive symptoms with age.

The findings of this study have clear clinical and practical significance. They support the implementation of a patient‐centered, multi‐target integrated intervention model. In clinical practice, it is recommended to conduct routine screening for depressive symptoms among arthritis patients, particularly targeting those with pronounced pain, lower education levels, or functional limitations, especially males and younger patients. Interventions should go beyond simple pharmacological pain management by integrating cognitive‐behavioral therapy to help patients reframe maladaptive cognitions about pain and their own functioning, using occupational therapy to maintain daily activity capacity and protect self‐efficacy, and enhancing digital health empowerment (e.g., providing age‐appropriate training for using health apps) to improve patients' ability to access information and social connection. This multi‐dimensional approach can build a more robust psychological protective network.

### 5.1 Limitations

Of course, this study has several limitations. First, the cross‐sectional design precludes causal inference between pain and depression; a complex bidirectional relationship is possible. Second, all data were based on self‐reports, which may introduce common method bias. Third, this study did not differentiate between specific subtypes of arthritis (e.g., osteoarthritis vs. rheumatoid arthritis), for which pain mechanisms and psychological impacts may differ. Future research should employ longitudinal designs, tracking pain, potential biomarkers (e.g., inflammatory markers), objective functional measures, and depressive symptoms across multiple time points to clarify causal pathways and mechanisms. Concurrently, qualitative studies to gain an in‐depth understanding of the psychological coping strategies of highly resilient patients, and to clarify the reasons behind the association between medical visits and more severe depressive symptoms, would further help optimize clinical support and communication strategies.

### 5.2 Clinical Implications for Practice

The findings of this study provide specific, actionable guidance for clinical practice. The core lies in shifting from universal management to a dual strategy of precise identification of high‐risk groups and strengthening protective resources. It is recommended to prioritize male patients, those aged <65, and those with lower educational levels who experience pain for depression screening and intervention. Intervention plans should be integrative, combining medical pain management with cognitive‐behavioral interventions to modify pain‐related maladaptive cognitions; utilizing occupational therapy for home environment assessments and activity modifications to help patients maintain meaningful functional engagement (e.g., housework); and providing age‐appropriate digital health skills training in community or clinical settings to empower patients to use the internet for support and information. Implementing this comprehensive care model that equally addresses physical, psychological, and social functioning can more effectively alleviate the burden of depression and holistically improve patients' quality of life.

## 6 Conclusion

In conclusion, depressive symptoms in arthritis patients are influenced by a complex interplay of multiple factors, including pain, sociodemographic characteristics, and functional status. Pain is a clear risk factor, while education, self‐rated health, housework ability, and internet use form an intervenable protective system. The finding that the pain‐depression association is more pronounced in specific subgroups (males, younger, and lower‐educated individuals) suggests that psychological interventions need to be individualized and precise. Future clinical practice and research should focus on developing and testing integrated intervention models that, while effectively managing pain, strive to enhance patients' psychosocial resources and functional independence, ultimately achieving comprehensive, patient‐centered health promotion.

## Author Contributions

**Limei Jiang**: conceptualization, investigation, funding acquisition, writing – original draft, resources, software. **Ming Tang**: conceptualization, investigation, software, data curation. **Yufan Zheng**: conceptualization, software, formal analysis. **Yao Zhang**: writing – original draft. **Jing Li**: writing – original draft. **Qingyue Kong**: software, supervision. **Fujie Jing**: writing – original draft, supervision, resources.

## Funding

This study was supported by the Postgraduate Quality Improvement and Innovation Project of Shandong University of Traditional Chinese Medicine (Grant Nos. YJSTZCX2024021, YJSTZCX2025046).

## Ethics Statement

Ethical approval for CHARLS was obtained from the Biomedical Ethics Review Committee of Peking University (IRB00001052‐11015), and no additional ethical approval was required for this study.

## Consent

Written informed consent was obtained from all participants before the interviews were conducted. Additionally, all patients were in full possession of their faculties and capable of understanding and willing, while patients with cognitive deficits were not included in the study.

## Conflicts of Interest

The authors declare no competing interests.

## Data Availability

The datasets used and/or analyzed during the current study are available from the corresponding author on reasonable request.
